# Supplementary material for: Influences of roughness and filling degree on the shear strength and damage evolution characteristics of cement-filled joints
Source: PLoS One. 2025 Jan 24;20(1):e0314229. doi: 10.1371/journal.pone.0314229 (PMC11761587; doi:10.1371/journal.pone.0314229)
Supplement: Attachment — Submitted filename: Response to Reviewers.docx [file pone.0314229.s002.docx]

Response to the review comments

**Journal:** PLOS ONE

**Manuscript Number:** PONE-D-23-44098

**Title:** Influences of roughness and filling degree on the shear strength and damage evolution characteristics of cement-filled joints

**Author(s)**: Zhilin Shu; Yang Liu; Yicheng Ye; Weiqi Wang; Binyu Luo; Jinpeng Jia

Dear Editor-in-Amirsalar Khandan, Ph.D. and Reviewers:

Thank you for your letter and for the reviewers’ comments concerning our manuscript entitled “Influences of roughness and filling degree on the shear strength and damage evolution characteristics of cement-filled joints”. Those comments are all very valuable and helpful for revising and improving our manuscript, as well as the important guiding significance to our further research. We have studied the comments carefully and have corrected them. We hope it will meet with your approval. The revised portions are marked **in red** in the manuscript. The main corrections in the manuscript and the responses to the reviewer’s comments are as flowing.

We appreciate editors-in- Amirsalar Khandan, Ph.D and the Reviewers’ warm work earnestly and hope that the correction will meet with approval.

Finally, thank you again sincerely for your valuable advice.

Best regards.

Yang Liu on behalf of all authors

---------------------------------------------------------------

Corresponding author:

Name: Yang Liu

Telephone: +86 135-9422-8774

E-mail: liuyang@wust.edu.cn

School of Resources and Environmental Engineering,

Wuhan University of Science and Technology,

Wuhan, 430081, P.R. China

---------------------------------------------------------------

Responds to the reviewer’s comments:

1. **What are the two main factors investigated in this study regarding cement-filled joints?**

**Response:** Thank you for your good suggestion. The main factors are roughness, filling and normal stress.

2.	**How was the roughness of the joints measured or characterized in the experiments?**

**Response:** Thanks for your valuable suggestion. Barton proposed the joint surface roughness standard, using JRC to quantitatively describe the surface roughness of rock joints, and this paper selects three of the ten standard roughness curves defined by it, and the roughness of the poured sample corresponds to the three standard curves.

3.	**What techniques were used to evaluate the damage evolution characteristics of the cement-filled joints under shear loading?**

**Response:** Thank you for your excellent suggestion. The whole shear process was monitored by acoustic emission technology, and the number of events, event locations and energy were obtained to evaluate the damage evolution characteristics of cement-filled joints under shear load.

4.	**Did the results show any differences in the shear strength and damage evolution between joints with different roughness and filling degrees? If so, what were the key findings?**

**Response:** Thanks for your good suggestion. There are differences in the shear strength and damage evolution of joints with different roughness and filling degrees, and the specific differences are as follows. The peak shear strength of the filled joint specimen was positively correlated with roughness and negatively correlated with the filling degree. During the shearing process, the damage degree of the filled joint specimen was positively correlated with the roughness, and negatively correlated with the filling degree.

**Other changes:**

We tried our best to improve the manuscript and made some changes in the manuscript. These changes will not influence the content and framework of the paper. And here we did not list the changes but marked them in red in the revised manuscript.
